# 50 Ω driver circuits for digital signals: Revised test board and new measurement data

**DOI:** 10.1016/j.dib.2023.110011

**Published:** 2023-12-26

**Authors:** Tilman Küpper

**Affiliations:** Hochschule München University of Applied Sciences, Department of Mechanical, Automotive and Aeronautical Engineering, Lothstr. 34, 80335 Munich, Germany

**Keywords:** Interface electronics, Line driver, Pulse generator, Trigger output

## Abstract

In “Evaluation of 50 Ω driver circuits for digital signals based on oscilloscope measurements” (Heliyon, Volume 7, Issue 7, July 2021) different 50 Ω driver circuits for digital signals were compared and evaluated. Notably, the BJT-based discrete push-pull driver circuit showed a rather modest performance. It is also questionable whether the fast-switching signals of the individual driver circuits were reproduced completely and correctly with the PicoScope 3206A oscilloscope used in that study, as its sampling rate is quite low.

For these reasons, the experiments were continued, several improvements were made to the test board. A new driver circuit was implemented on the test board, too. New measurement data were recorded both with a Tektronix MDO3024 oscilloscope and for comparison with the previously used PicoScope 3206A.

The following dataset is made available: The revised test board (PCB layout and schematic design files), oscilloscope measurement data in CSV format, Python scripts for graphical representation of the CSV data.

Specifications Table

**Table:** 

Subject	Electrical and electronic engineering
Specific subject area	Driver circuits for digital signals
Data format	Raw, Analyzed
Type of data	Table, Image, Graph, Source Code
Data collection	The test board was designed using KiCAD, version 6. Measurements were conducted using Tektronix MDO3024 and PicoScope 3206A oscilloscopes, the results were saved in CSV format. Graphical representations of the signal waveforms were generated from the CSV data using Python scripts.
Data accessibility	Tilman Küpper: 50 Ω driver circuits for digital signals: Revised test board and new measurement data. Mendeley Data, 2023. [2] https://doi.org/10.17632/zmyn93k6bw.1
Related research article	Tilman Küpper: Evaluation of 50 Ω driver circuits for digital signals based on oscilloscope measurements. Heliyon, Volume 7, Issue 7, July 2021. [1] https://doi.org/10.1016/j.heliyon.2021.e07674

## 1 Value of the Data

• Manufacturers of electronic components document a large amount of technical data in their data sheets, but rarely measurements on real built circuits. This gap is filled by the measurement data documented in [1] and in this article. All driver circuits are documented in detail, including the respective PCB layout. All design files and measurement data are freely available on Mendeley Data [2]. These data are relevant for all users of the components shown here, including for the development of other types of circuits.
• In [1], 50 Ω driver circuits for digital signals are compared and evaluated. The performance of the BJT-based discrete push-pull driver is not convincing. For the other circuits, the question arises whether they can be further optimized. The experiments were therefore continued, and a revised test board was developed. This article is the continuation of the work documented in [1]. It is relevant for other research work in the field of circuit technology, especially if high-speed switching applications are involved.
• The revised new driver circuits are also fully documented with PCB layout, schematic and oscilloscope measurement data. The driver circuits documented here can also serve as a basis for further research work. This makes them easy to use as building blocks for new designs and useful for other electronics designers and researchers.
• In addition, the test board presented here can also be used directly as a pulse generator in laboratory experiments. As shown in [1], some of the driver circuits on the test board have faster rise and fall times than many commercial function generators. Compared to the original version, an auxiliary interface has been added. Buttons or LEDs can be connected to this interface, making the test board quite flexible to use for many potential applications, for example in university teaching or as part of research projects.

## 2 Background

50 Ω driver circuits for digital signals are rarely dealt with in the literature as well as on the internet. Therefore, the idea was born to build a test board with different driver circuit variants, to evaluate them systematically and to compare them. First results were documented in [1].

On the original test board, the performance of the BJT-based discrete push-pull driver is not convincing. For the other driver circuits, the question arises whether they can be further optimized. The experiments were therefore continued, and a revised test board was designed following literature and internet research. A new driver circuit, based on TLV3501 high-speed comparators, is included on the revised test board. In addition, to further improve the switching characteristics of all driver circuits, a smaller footprint was selected for the ceramic blocking capacitors. Further details can be found in “Experimental design, materials and methods”.

The oscilloscope measurement data in [1] were acquired with a PicoScope 3206A USB oscilloscope. The sample rate of this oscilloscope is quite moderate at 500 MS/s. It has to be questioned, whether the fast-switching signals of the individual driver circuits were reproduced completely and correctly with this oscilloscope. The measurement data on the revised test board were therefore acquired with a Tektronix MDO3024 oscilloscope with a sampling rate of 2.5 GS/s, and repeated with the PicoScope 3206A for comparison.

## 3 Data Description

Table 1 provides an overview of the contents of the dataset available on Mendeley Data.

**Table 1:** Contents of the dataset.

testboard.zip	KiCAD design files including PCB layout and schematic of the test board
tektronix-data.zip	Raw measurement data in CSV format, acquired with Tektronix MDO3024 oscilloscope
tektronix-figures.zip	Figures generated from Tektronix MDO3024 measurement data
picoscope-data.zip	Raw measurement data in CSV format, acquired with PicoScope 3206A oscilloscope
picoscope-figures.zip	Figures generated from PicoScope 3206A measurement data
firmware.zip	ATtiny2313 microcontroller firmware used on the test board

Schematics and PCB layout of driver circuits 1, 2, 4, 5 and 6 are documented in [1]. They can also be found in **testboard.zip**. See section “Experimental design, materials and methods” for details on the newly developed driver circuit “Test 3N” (TLV3501 high-speed comparator) and other modifications to the test board. The revised PCB layout is shown in Fig. 6.

An ATtiny2313 microcontroller generates the test signals for the individual driver circuits on the test board. **firmware.zip** contains the firmware running on the microcontroller, it has not changed compared to the original version.

All measurements on the revised test board were performed with two different oscilloscopes. In addition to the PicoScope 3206A used in [1], the measurements were repeated with a Tektronix MDO3024 for comparison. The raw measurement data in CSV format can be found in **picoscope-data.zip** and **tektronix-data.zip**.

**Fig. 1:**
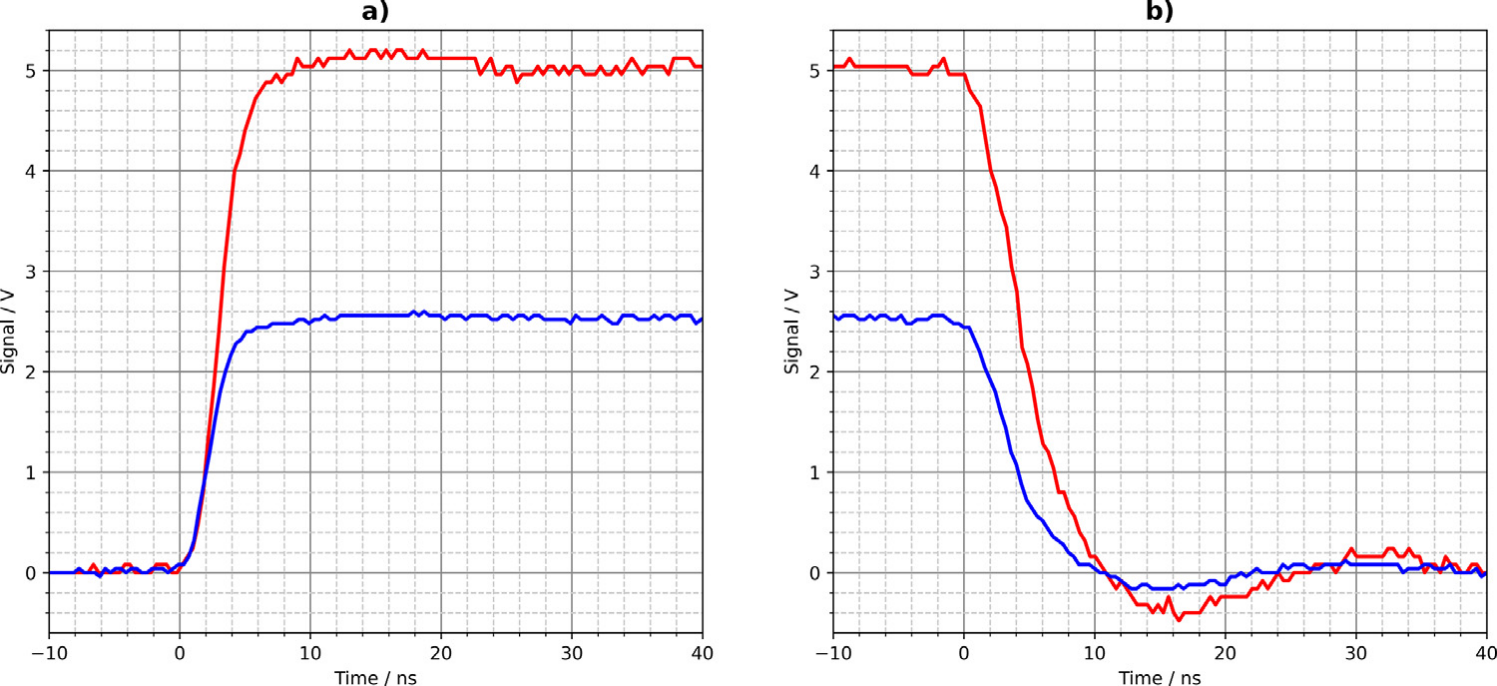
Driver circuit 1, parallel port pins: a) rising edge, b) falling edge.

**Fig. 2:**
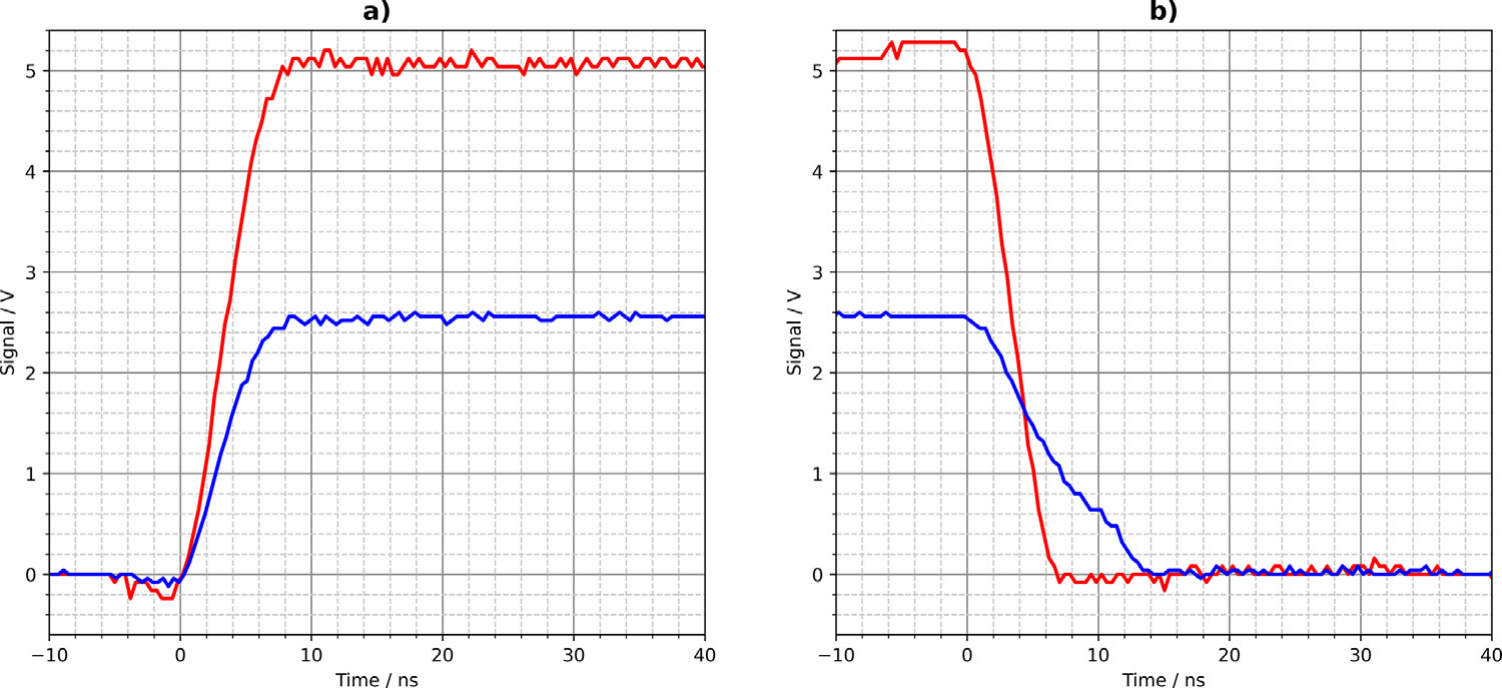
Driver circuit 2, MCP14A0602 MOSFET gate driver: a) rising edge, b) falling edge.

**Fig. 3:**
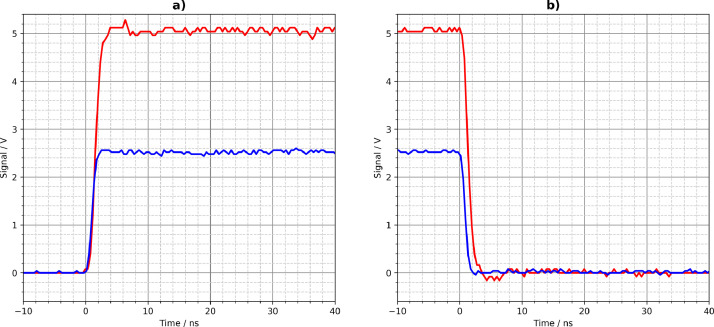
Driver circuit 3N, TLV3501 high-speed comparator: a) rising edge, b) falling edge.

**Fig. 4:**
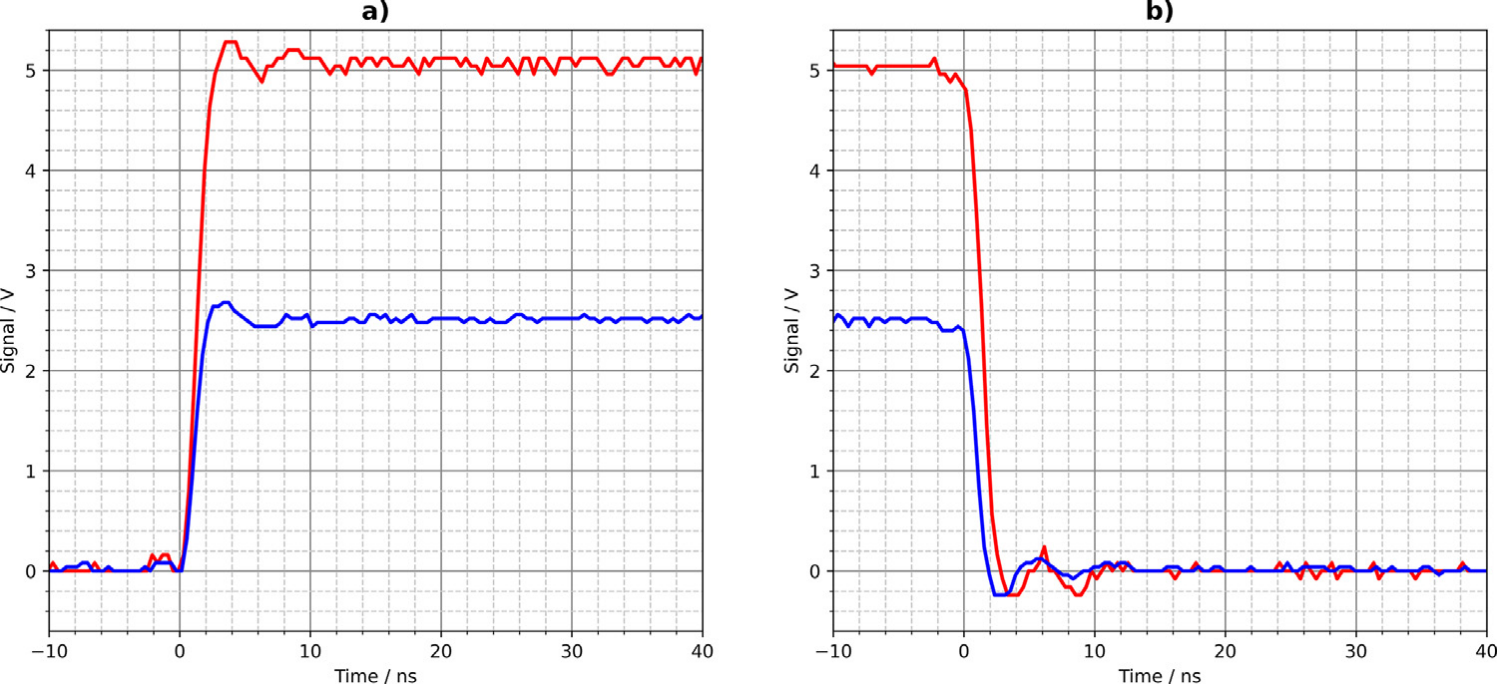
Driver circuit 4, parallel 74LVC1G04 logic gates: a) rising edge, b) falling edge.

**Fig. 5:**
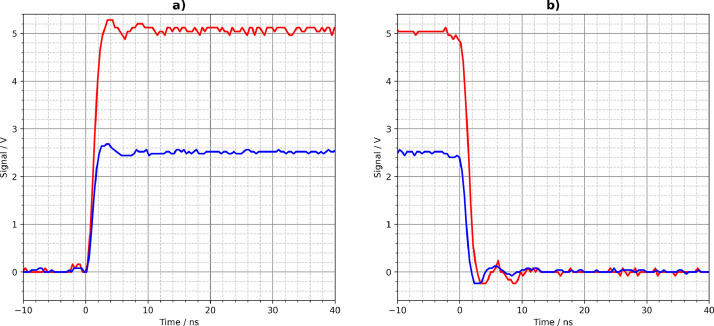
Driver circuit 5, parallel CD74AC04M logic gates: a) rising edge, b) falling edge.

Fig. 1, Fig. 2, Fig. 3, Fig. 4, Fig. 5 show the Tektronix MDO3024 measurement results of all driver circuits on the test board. They were generated from the CSV files using Python scripts. All figures and Python scripts can be found in **picoscope-figures.zip** and **tektronix-figures.zip**, respectively. The blue signals were recorded with a 50 Ω load resistor, the red signals without load (open output). This applies to all figures in this section.

## 4 Experimental Design, Materials and Methods

The research article “Evaluation of 50 Ω driver circuits for digital signals based on oscilloscope measurements” [1] was published in Heliyon, Volume 7, Issue 7, July 2021. Different 50 Ω driver circuits for digital signals proposed in the literature and on the Internet were systematically compared and evaluated. The aim was to create a starting point for subsequent work, which did not exist in this form.

All driver circuits were built and evaluated on the same four-layer PCB with the following layer stackup: L1 - signals, L2 - GND plane, L3 - VCC plane, L4 - signals. As all driver circuits are placed on the same test board, their performance can be compared directly with each other. A four-layer PCB was chosen to achieve optimal dynamic characteristics of the driver circuits. The test board presented here has solid GND and VCC planes. This reduces noise and EMI effects and contributes to cleaner and more reliable signal transmission ([11], p. 697). The distribution of traces and components over several layers also enables shorter signal paths.

**Fig. 6:**
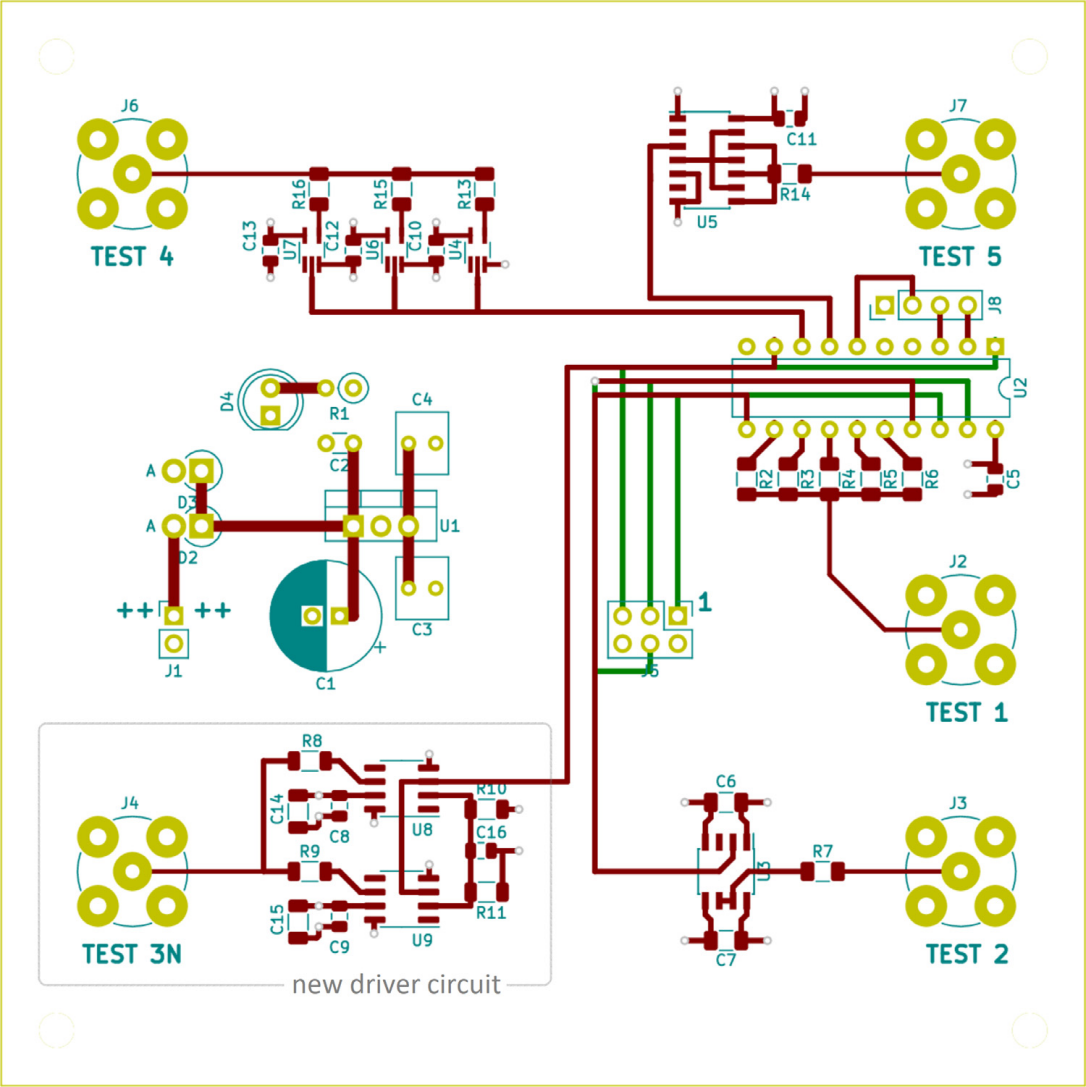
Revised PCB layout of the test board.

Fig. 6 shows the PCB layout of the revised test board. The complete schematic can be found in the dataset [2] in testboard.zip. At the center of the test board there is an ATtiny2313 microcontroller (Fig. 6, U2). It operates at a clock rate of 8 MHz. The supply voltage of 5 volts is provided by a linear voltage regulator (U1). The microcontroller generates the trigger signals for the individual driver circuits. The microcontroller firmware ensures that the driver circuits are not triggered simultaneously, but one after the other. This avoids mutual interference and ensures clean measurements. Firmware updates can be flashed to the microcontroller at any time via the J6 connector.

The clock frequency of the microcontroller is less relevant for the tests carried out here, however. The purpose of the various driver circuits is rather to generate fast-switching and clean digital trigger signals in a simple way. As the oscilloscope measurements show, rise times and fall times of a few nanoseconds are possible with the driver circuits evaluated here. This is achieved without any significant overshoot or undershoot, regardless of whether 50 Ω load resistors are connected or not (open output).

The high-level output voltage of 5 V (no load) combined with an output impedance of 50 Ω makes the driver circuits quite flexible to use. It is equally possible to feed transmission lines (e. g. coaxial cables) as well as connect various types of digital inputs of subsequent components. See [1] for further details.

## 5 Improvements to the Original Version of the Test Board

Notably, the BJT-based discrete push-pull driver circuit “Test 3” on the original version of the test board exhibited a rather modest performance. The oscilloscope measurements on the other driver circuits have already shown promising results. Nevertheless, the question arises as to whether they can be further optimized. To address this, several refinements were made to the test board.

**Fig. 7:**
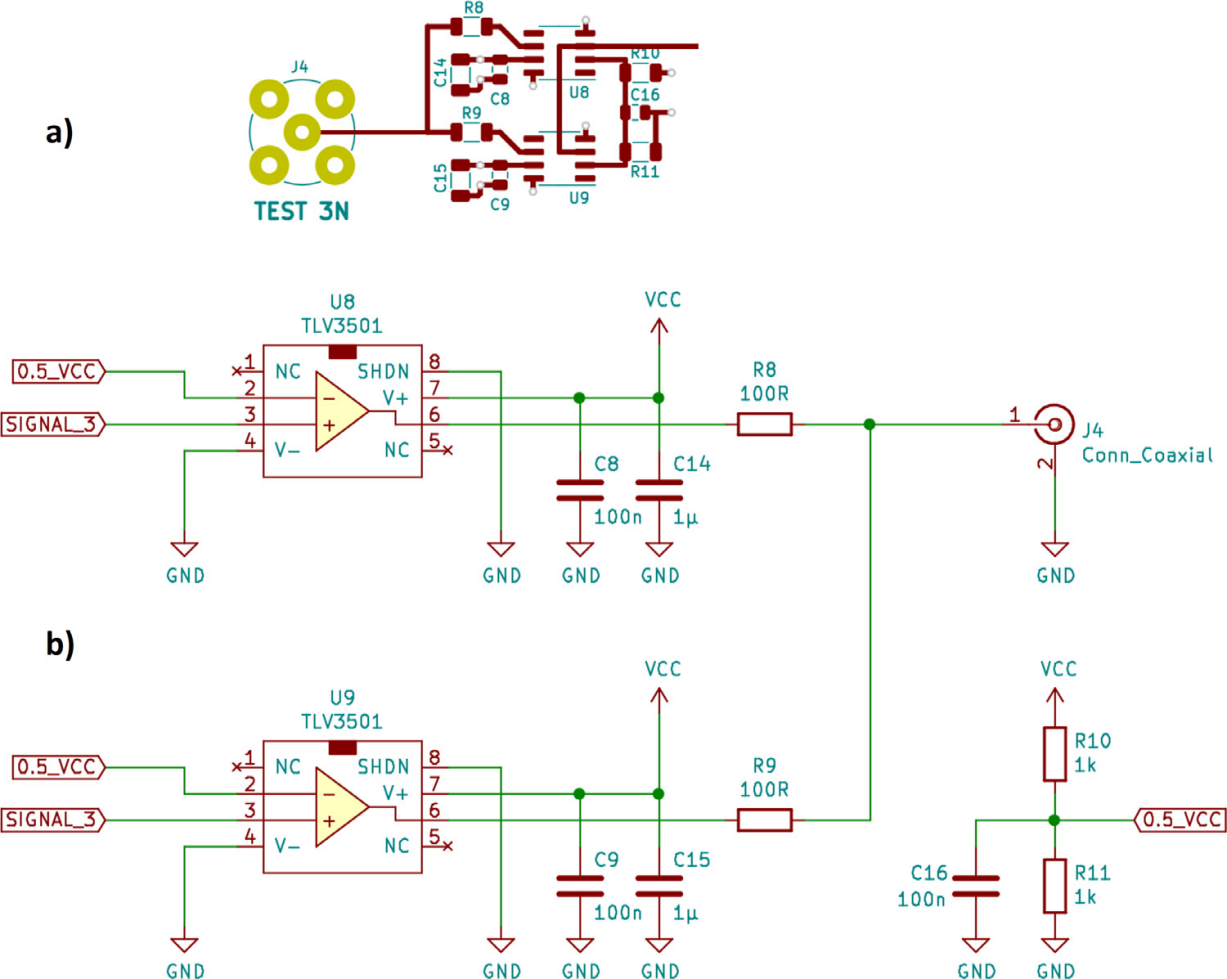
TLV3501-based driver circuit “Test 3N”, a) PCB layout, b) schematic.
• To further improve the switching characteristics of all driver circuits on the test board, a more compact 0805 footprint was selected for all 100 nF ceramic capacitors instead of the 1206 footprint previously used. As indicated in [6] (Figure 1x.58) and [7] (Section 23.1.3), the interfering parasitic inductance (ESL) decreases with the smaller component size.
• As already mentioned in [1], the test board presented here can also be used directly as a pulse generator in laboratory experiments. Typical examples are time-domain reflectometry (TDR) measurements, where signals with very short rise and fall times are required. Compared to the original version, an auxiliary interface has been added (see Fig. 6, connector J8). Pushbuttons, LEDs, and other components can be connected to this interface, making the test board more flexible to use.
• The original version of the test board was developed using KiCAD schematic and PCB layout software, version 5. This version is no longer supported. The original design files were therefore converted to KiCAD, version 6 [10]. All modifications documented in this article were then implemented in version 6.

• Specifically, “Test 3” was replaced with a new driver circuit “Test 3N” based on the high-speed TLV3501 rail-to-rail comparator [8]. Comparator-based driver circuits were not yet implemented on the original test board. A comparator is a high-gain differential amplifier that compares two analog input signals. The voltage at the output of the comparator only takes two possible values (“high” or “low”), depending on which of the two input voltages is higher. Comparators are normally used to classify analog sensor signals, to monitor battery voltages or to build certain types of oscillator circuits.
• Some common comparators are listed in [6] (Table 12.1, p. 812), with the TLV3501 (Texas Instruments) featuring the fastest switching time (4.5 ns, typ.). Its output stage based on a complementary transistor pair can switch at high speed and at the same time deliver significant current. It was therefore selected as the basis for the new driver circuit on the test board. The resistors R8 and R9 provide the desired output impedance of 50 Ω as well as the required short-circuit protection: Due to the parallel connection of two TLV3501s, the output current always remains below 50 mA per component (absolute maximum rating according to the datasheet [8]: 74 mA), even with the output of the driver circuit shorted.
• The TLV3501 has also been employed successfully in other research projects. For example, this component is used in [3] to capture signals on a CAN bus and feed them to an FPGA for further processing. A high frequency, high power density, and high efficiency relaxation oscillator in [4] is also based on the TLV3501. Finally, the same comparator is used in [5] as part of a pulse generator for a high-frequency ultrasound system. These examples illustrate the universal applicability of the TLV3501.
• Schematic and PCB layout of the new TLV3501-based driver circuit “Test 3N” are shown in Fig. 7. See Fig. 3 for the corresponding oscilloscope measurement results.

**Fig. 8:**
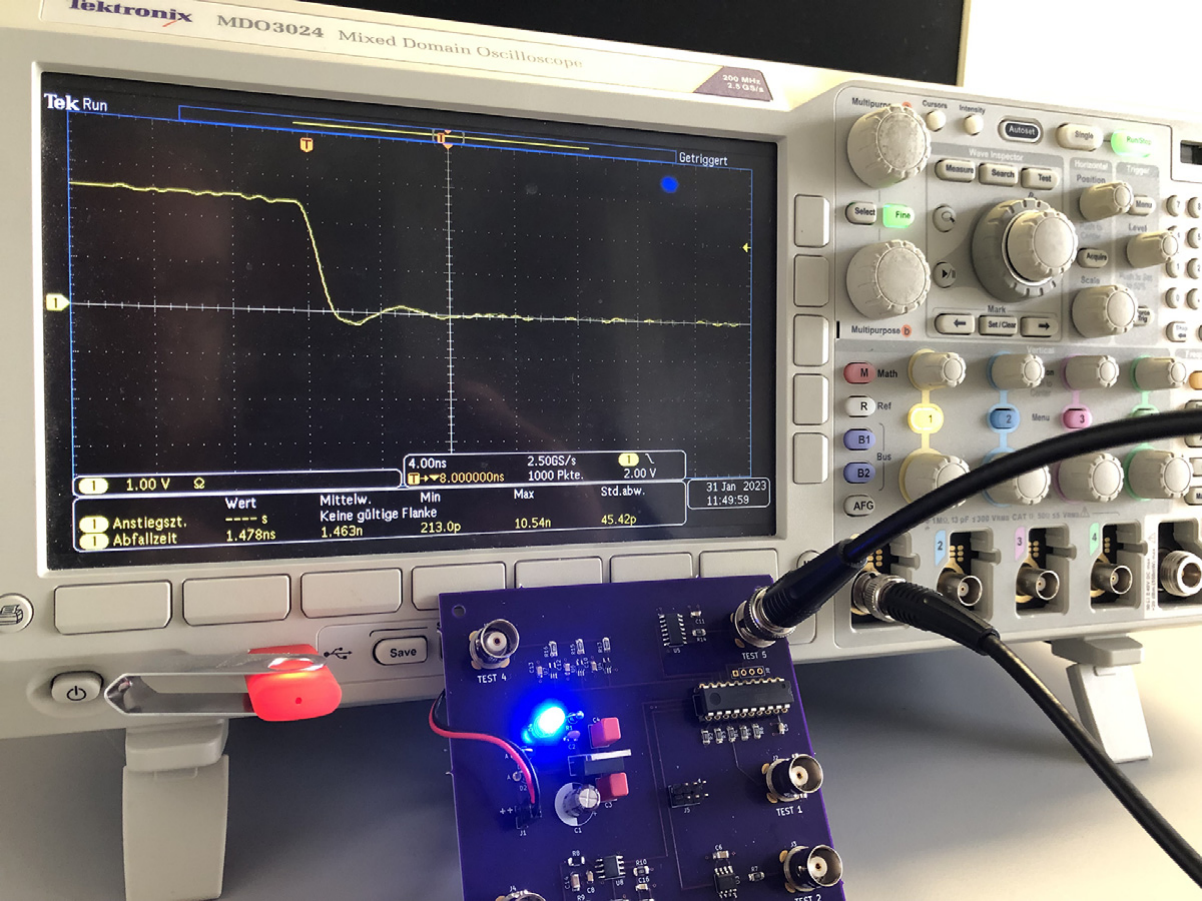
Tektronix MDO3024 oscilloscope measurements.

The measurements on the revised test board were performed with two different oscilloscopes. In addition to the PicoScope 3206A used in [1], all measurements were repeated with a Tektronix MDO3024 for comparison. Fig. 8 shows the revised test board connected to the MDO3024, a 200 MHz bandwidth, 2.5 GS/s, four-channel oscilloscope. Detailed specifications of this oscilloscope can be found on the manufacturer's website [9].

These comparative measurements make it easier to assess the validity of the documented measurement results. Dependencies of the measurement results on the oscilloscope used can be traced from the associated data set and can also be estimated by comparing them with the results published in [1].

## Limitations

Not applicable.

## Ethical Statement

The author has reviewed and is in full compliance with the ethical requirements for publication in Data in Brief. The current work does not involve human subjects, animal experiments, or any data collected from social media platforms.

## Credit Author Statement

Tilman Küpper: Conceptualization, Methodology, Software, Investigation, Visualization, Writing - original draft, Writing - review & editing.

## Data Availability

50 Ω driver circuits for digital signals: Revised test board and new measurement data (Original data) (Mendeley Data)
